# Circulating cytokines levels and the risk of polycystic ovary syndrome: A Mendelian randomization analysis

**DOI:** 10.1097/MD.0000000000041359

**Published:** 2025-02-28

**Authors:** Yumin Jiang, Yunqing Li, Yuhua Huang

**Affiliations:** a Graduate School, Beijing University of Chinese Traditional, Beijing, China; b Gynecology Department, Beijing Traditional Chinese Medicine Hospital Affiliated to Capital Medical University, Beijing, China.

**Keywords:** cytokinesis, inflammation, Mendelian randomization analysis, polycystic ovary syndrome

## Abstract

This study utilized Mendelian randomization (MR) analysis to explore the causal relationship between circulating cytokines and polycystic ovary syndrome (PCOS), and to identify potential biomarkers of PCOS mechanisms. Genetic instrumental variables for cytokines were derived from 2 large-scale genome-wide association studies (GWAS) involving 8293 and 14,824 European participants. Summary statistics from a GWAS meta-analysis (10,074 PCOS cases and 103,164 controls of European ancestry) were used in the discovery phase of MR analysis. Replication analysis utilized another GWAS meta-analysis dataset (3609 cases and 229,788 controls). The primary analysis employed the inverse-variance weighted (IVW) method, with secondary methods including constrained maximum likelihood model averaging, weighted median, and weighted mode. Meta-analysis was combined with MR results, while heterogeneity and horizontal pleiotropy were assessed using leave-one-out, MR-Egger intercept test, and Mendelian Randomization Pleiotropy Residual Sum and Outlier. Sensitivity analysis confirmed the robustness of the results. Reverse MR analysis was used to explore the association of PCOS with the identified cytokines. Meta-analysis revealed that increased CCL4 (C-C motif chemokine 4) levels were associated with a higher risk of PCOS (odds ratio [OR] = 1.123, 95% confidence interval [CI]: 1.056–1.195; *P* < .001). Decreased PCOS risk was linked to CXCL11 (C-X-C motif chemokine 11, OR = 0.930, 95% CI: 0.890–0.970; IVW-false discovery rate [FDR] *P* = 4.85 × 10^−4^) and CD6 (T-cell surface glycoprotein CD6 isoform, OR = 0.730, 95% CI: 0.890–0.970; IVW-FDR *P* = .008). Sensitivity analysis confirmed the robustness of the findings. MR analysis suggests a potential causal link between alterations in CCL4, CXCL11, CD6, and PCOS risk, highlighting the role of cytokines in PCOS development and progression, warranting further investigation.

## 1. Introduction

Despite affecting approximately 11–13% of women globally, research on polycystic ovary syndrome (PCOS) remains insufficient. The total annual healthcare-related economic burden of PCOS surpasses $15 billion in the United States alone, encompassing expenses for PCOS diagnosis and those linked to mental health disorders, reproductive issues, vascular complications, and metabolic disorders associated with PCOS. Reports indicate that among women of reproductive age worldwide, 1.55 million cases of were diagnosed, leading to 0.43 million (ranging from 0.19 to 0.82 million) disability adjusted life years linked to the ailment, thus highlighting the significant global economic burden. PCOS, which is characterized by insulin resistance and hyperandrogenism, contributes to the early onset of type 2 diabetes and increases the risk of cardiovascular disease. The reproductive implications of PCOS include irregular menstrual cycles, infertility, increased risk of pregnancy complications, and higher risk of endometrial cancer.^[1]^

Current research on potential risk factors associated with PCOS is comprehensive and continuously advancing. Established risk factors include genetic predisposition,^[2]^ excessive androgen levels,^[3]^ and metabolic dysregulation.^[4]^ Evidence suggests that inflammation plays a role in the onset and development of PCOS.^[5]^ Inflammation is a common link between obesity, cardiovascular disease, insulin resistance, and diabetes,^[6]^ all of which are metabolic manifestations of PCOS. Observational studies have demonstrated dysregulation of several cytokine levels in the circulation of patients with PCOS.

For example, previous epidemiological studies have shown elevated circulating levels of specific cytokines in women with PCOS compared with age- and BMI-matched controls. For instance, monocyte chemoattractant protein-1,^[7]^ interleukin-10,^[8]^ interleukin-6, and tumor necrosis factor-α levels have been found to be higher,^[9]^ whereas interleukin-10,^[8]^ leptin, and adiponectin levels are lower.^[10]^

However, studies on the association between specific cytokines and PCOS risk have yielded conflicting results. For example, 1 case-control study reported higher interleukin-18 levels in PCOS patients than in controls, whereas another study reported no significant differences. Similar contradictions have also been observed in studies on interleukin-11, which may arise from the interplay of inflammation, obesity, hyperandrogenism, and insulin resistance in the pathogenesis of PCOS. Considering the potential role of increased androgen levels in triggering inflammation and disrupting cytokine regulation, these findings may exhibit reverse causal tendencies.

Notably, a significant portion of the evidence regarding PCOS stems from observational studies that are limited by various biases and unmeasured confounding factors. Randomized controlled trials remain the gold standard for etiological inference because they can address the primary limitations of observational studies. However, considering the practical drawbacks of randomized trials, including high financial costs and extended timeframes, Mendelian randomization (MR) methods offer a viable and robust alternative for inferring causality.

MR is less affected by reverse causation and potential environmental and social confounding factors. MR uses genetic variants such as single nucleotide polymorphisms (SNPs), which are strongly associated with exposure, as instrumental variables (IVs) to establish causal relationships. Our study conducted a bidirectional 2-sample MR analysis to examine the association between the predicted genetic risk of cytokines and PCOS, aiming to mitigate biases and confounding factors inherent in traditional studies and to provide novel insights for future treatment and prevention strategies.

## 2. Materials and methods

### 2.1. Study design

**Figure 1.**
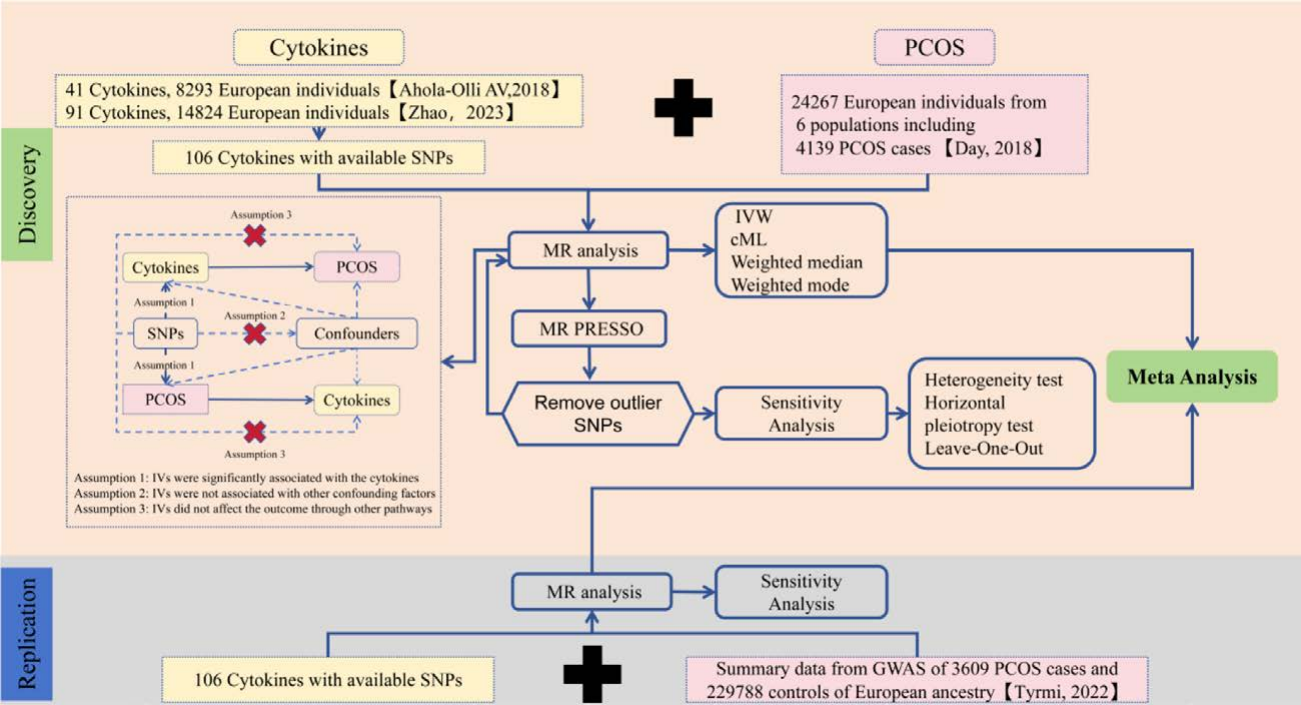
Study design and workflow of this study.

The overall design of the bidirectional 2-sample MR study is illustrated in Figure 1. To ensure the credibility of our findings derived from the MR approach, we aimed to satisfy 3 fundamental assumptions inherent to MR studies. First, the IVs demonstrated significant associations with circulating cytokine levels. Second, the IVs were not correlated with other potential confounders. Finally, in addition to exposure factors, IVs must not influence outcomes through alternative pathways. Using publicly available genome-wide association study (GWAS) summary statistics, no additional ethical approval was required.

### 2.2. Data source and instruments

#### 2.2.1. Cytokines

The cytokine data for this study were obtained from 2 GWAS datasets (Table S1, Supplemental Digital Content, http://links.lww.com/MD/O384). Recent research has investigated the correlation of 41 cytokines with diseases, adjusting for age, sex, body mass index (BMI), and the first ten genetic principal components.^[11,12]^ However, the latest GWAS data have identified up to 91 cytokines associated with inflammation adjusted for age, sex, and other study-specific covariates.^[13]^ To encompass a broader range of cytokines, we initially selected GWAS data for 91 inflammation-related cytokines from 11 cohorts comprising 14,824 European individuals. Additionally, data from 8293 European individuals across 8 cohorts were included for 15 non-overlapping cytokines (Table S2, Supplemental Digital Content, http://links.lww.com/MD/O384).

In the primary analysis, cytokines with independent SNPs were identified using a *P* value threshold of <5 × 10^−8^. Owing to the limited number of cytokines meeting genome-wide significance, the threshold was adjusted to <5 × 10^−6^. Linkage disequilibrium analysis (LDA) ensured SNP independence, with criteria set at *r*^2^ < 0.001 and distance = 250 kb.^[14]^

#### 2.2.2. Polycystic ovary syndrome

PCOS data were extracted from the largest GWAS database,^[15]^ encompassing 25,295 participants of European descent, including 4890 patients with PCOS (Table S1, Supplemental Digital Content, http://links.lww.com/MD/O384). In 2018, data were collected from 7 cohorts using 3 diagnostic methods: self-reported diagnosis of PCOS, and diagnoses based on the NIH or non-NIH Rotterdam criteria. Further details are available in the original publications. A *P* value threshold of <5 × 10^−8^ was applied to identify PCOS-associated SNPs, with concurrent LDA performed to ensure SNP independence (*r*^2^ < 0.001 and distance = 10,000 kb). To enhance the robustness of the study and mitigate false positives, an additional dataset of 233,397 individuals, including 3609 patients with PCOS, was sourced from the GWAS database.^[16]^ In 2022, Tyrmi et al employed ICD codes (ICD-10 E28.2, ICD-9 256.4, or ICD-8 256.90) from national registries to identify PCOS cases with all other females serving as controls. A meta-analysis was performed on 3609 cases and 229,788 controls from 2 cohorts using data collected from the FinnGen study and an independent dataset from the Estonian Biobank. Cohorts collected by Tyrmi et al and Day et al did not overlap. Similar to the primary analysis, a *P* value threshold of <5 × 10^−8^ was applied to identify PCOS-related SNPs, and LDA was conducted to confirm SNP independence, using criteria of *r*^2^ < 0.001 and distance = 10,000.

All genetic data were obtained from publicly available GWAS with prior ethical approval and participant consent, thus obviating the need for additional ethical reviews.

### 2.3. Statistical analysis

After extracting the SNP data for cytokines, SNPs strongly associated with PCOS and known confounding factors were excluded (Table S3, Supplemental Digital Content, http://links.lww.com/MD/O384). Additionally, SNPs with an *F* statistic ≤ 10 were removed to evaluate the strength of the IVs (*F* > 10 indicates adequate strength). Ultimately, 1935 SNPs were selected as the IVs in this study (Table S4, Supplemental Digital Content, http://links.lww.com/MD/O384).

Bidirectional Mendelian randomization (MR) analyses were performed using the “TwoSampleMR” software package, with inverse-variance weighting (IVW) employed as the primary method owing to its precision and absence of bias. IVW is the most commonly used method for causal estimation in 2-sample MR analyses, particularly for robust IV validity. Therefore, IVW was selected as the primary and most effective analytical approach in this study. A random-effects IVW model was used in cases of statistically significant heterogeneity across studies; otherwise, a fixed-effects IVW model was used.

Additionally, we used constrained maximum likelihood with model averaging, and weighted median and mode analyses were applied to identify and mitigate potential confounders.

CML-MA is an MR analysis technique that effectively manages type I errors and improves testing efficiency compared with MR-Egger, particularly in addressing weak instrument bias. The weighted median method was employed for sensitivity analysis, offering robust estimates when up to 50% of the weights originated from valid IVs. The weighted mode method assumes that the most common causal effect estimate aligns with the true causal effect estimate, allowing for the dismissal of the other variables. By calculating the weighted average of each genotype, we comprehensively assessed the impact of different genotypes on phenotypes, thereby controlling for the influence of genotype frequency differences on the analysis results and enhancing the robustness and accuracy of the analysis.

*P* values were adjusted for the false discovery rate (FDR) using the Benjamini–Hochberg method. A *P* value below .05 after FDR correction indicated a statistically significant correlation. The results of both the discovery and replication MR analyses were combined for meta-analysis. A combined *P* value below .05 was considered statistically significant, with conclusions based on odds ratios (ORs).

### 2.4. Sensitivity analysis

To evaluate the robustness of our results, we tested for heterogeneity and pleiotropy levels, and performed leave-one-out (LOO) sensitivity analysis using the “TwoSampleMR” and “MRPRESSO” software packages in R (version 4.2.0).^[17]^

Initially, the Mendelian Randomization Pleiotropy Residual Sum and Outlier (MR-PRESSO) method was used to detect horizontal pleiotropy. Upon detection, outliers were eliminated for subsequent MR analysis. MR-Egger regression was employed to assess directional pleiotropy, with *P* values < .05 considered indicative of pleiotropy. In the absence of detected horizontal pleiotropy or outliers, Cochran’s *Q* test was used, where a *P* value > .05 indicated no heterogeneity. LOO analysis was performed to evaluate the impact of each SNP and identify any outliers.

## 3. Results

### 3.1. Discovery on the risk of PCOS

After eliminating redundant cytokines, the analysis included 106 unique cytokines from various sources (Tables S1, Supplemental Digital Content, http://links.lww.com/MD/O384 and S2, Supplemental Digital Content, http://links.lww.com/MD/O384). We extracted SNP data for cytokines using a significance threshold of *P* < 5 × 10^−6^ and excluded SNPs that were strongly associated with PCOS and known confounding factors (Table S3, Supplemental Digital Content, http://links.lww.com/MD/O384). Additionally, SNPs with an *F* statistic ≤ 10 were removed to ensure adequate strength of the IVs (*F* > 10 indicates adequate strength). Ultimately, 1927 SNPs were selected as the IVs in this study (Table S4, Supplemental Digital Content, http://links.lww.com/MD/O384).

**Figure 2.**
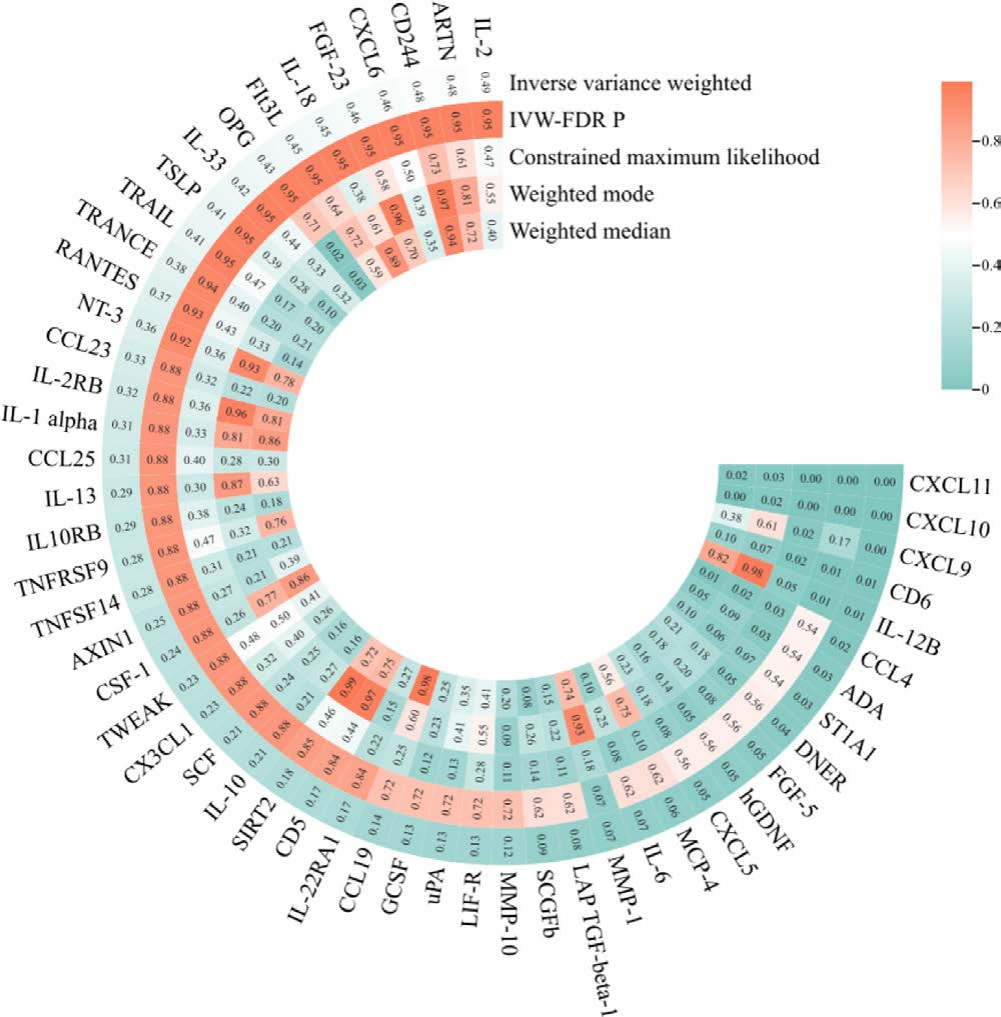
Heatmap of different MR analysis methods for cytokines and the risk of PCOS. MR = Mendelian randomization, PCOS = polycystic ovary syndrome.

In an MR analysis focusing on 106 cytokines and PCOS, the IVW method identified potential associations between the 10 cytokines and PCOS (Table S5, Supplemental Digital Content, http://links.lww.com/MD/O384; Fig. 2). Following FDR adjustment, elevated levels of CXCL10 (OR = 0.694, 95% confidence interval [CI]: 0.579–0.833, IVW-FDR *P* = 9.00 × 10^−5^), CXCL11 (OR = 0.657, 95% CI: 0.549–787, IVW-FDR *P* = 4.85 × 10^−4^), CD6 (OR = 0.762, 95% CI: 0.624–0.931, IVW-FDR *P* = .008), and IL-12B (OR = 0.887, 95% CI: 0.809–0.973, IVW-FDR *P* = .011) exhibited negative associations with PCOS. However, no significant positive association with PCOS risk was found. The concordance among the constrained maximum likelihood with model averaging, weighted median, and weighted mode methods with the IVW trend in the MR analysis underscores the robustness of these findings, with the exception of IL-12B.

In the sensitivity analysis, in addition to CXCL10, no SNP pleiotropy was detected in the pleiotropy and MR-PRESSO analyses (Table S6, Supplemental Digital Content, http://links.lww.com/MD/O384), indicating the robustness of IVs. Furthermore, the heterogeneity test showed no heterogeneity in the MR results (Table S7, Supplemental Digital Content, http://links.lww.com/MD/O384), further validating the reliability of our findings. The LOO method did not identify any significant bias points (Fig. S1, Supplemental Digital Content, http://links.lww.com/MD/O383).

In the MR analysis assessing the potential causal relationships between PCOS traits and cytokines, no such relationships were confirmed after FDR correction (Table S8, Supplemental Digital Content, http://links.lww.com/MD/O384). These results highlight the complex relationship between PCOS and cytokine levels. Overall, our findings are robust and suggest that multiple cytokines may be causally associated with PCOS.

### 3.2. Replication results of the risk of PCOS

**Figure 3.**
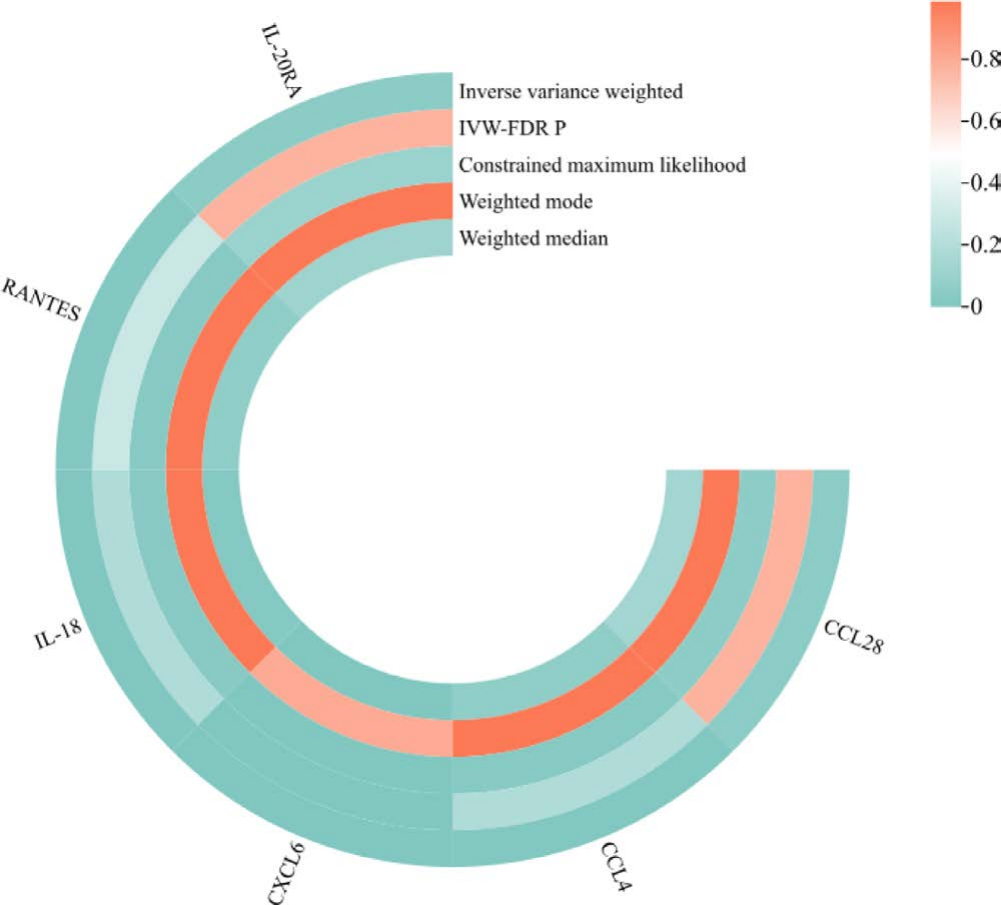
Heatmap of different MR analysis methods for cytokines and the risk of PCOS in replicate analysis. MR = Mendelian randomization, PCOS = polycystic ovary syndrome.

Similar to the discovery analysis, GWAS data related to PCOS were extracted from the database, encompassing 1738 robust SNPs (*F* > 10) and 106 cytokines (Tables S1 and S9, Supplemental Digital Content, http://links.lww.com/MD/O384). MR analysis identified associations of CCL28 (OR = 0.781, 95% CI: 0.615–0.993, *P* = .044), interleukin-18 (OR = 0.824, 95% CI: 0.723–0.940, *P* = .004), and RANTES (OR = 0.797, 95% CI: 0.669–0.949, *P* = .011) with PCOS risk. CCL4 (OR = 1.131, 95% CI: 1.097–1.233, *P* = .005) and CXCL6 (OR = 1.313, 95% CI: 1.160–1.489, IVW-FDR *P* = 1.82 × 10^−5^) were associated with an increased risk of PCOS. However, after adjustment, only CXCL6 [IVW-FDR, *P* = .002] showed a significant causal relationship with PCOS risk (Table S10, Supplemental Digital Content, http://links.lww.com/MD/O384; Fig. 3).

Horizontal pleiotropy testing and MR-PRESSO revealed no heterogeneity or pleiotropy (Table S11, Supplemental Digital Content, http://links.lww.com/MD/O384). Homogeneity tests of MR results showed no evidence of heterogeneity (Table S12, Supplemental Digital Content, http://links.lww.com/MD/O384). Furthermore, the LOO analysis confirmed the robustness of these findings (Fig. S2, Supplemental Digital Content, http://links.lww.com/MD/O383). Reverse MR analysis failed to demonstrate any causal relationship between PCOS risk and cytokine level (Table S13, Supplemental Digital Content, http://links.lww.com/MD/O384).

#### 3.2.1. Combined results of PCOS risk from the meta-analysis

**Figure 4.**
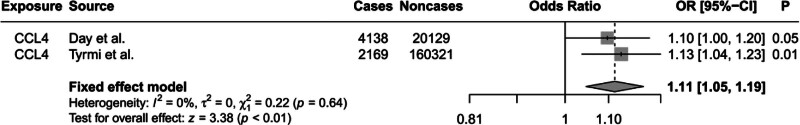
Forest map of combined results of PCOS risk from the meta-analysis. PCOS = polycystic ovary syndrome.

Our meta-analysis revealed a significant positive causal association between CCL4 (OR = 1.123, 95% CI: 1.056–1.195, *P* < .001) and PCOS (Fig. 4).

## 4. Discussion

In this study, we used a dual-sample MR method to assess the potential causal link between circulating levels of 106 cytokines and PCOS risk. Using primary and sensitivity analysis methods, we identified correlations between alterations in circulating levels of CXCL11, CD6, and CCL4, and the risk of PCOS. Additionally, we noted suggestive links between circulating levels of CXCL10 and IL-12B and risk of PCOS.

PCOS is a highly complex reproductive endocrine disease characterized by a multifaceted and intricate pathogenesis. Increasing evidence has underscored the significance of cytokines in the onset and progression of PCOS. Cytokines, which are signaling proteins produced by specific immune cells, influence various other cells.^[18]^ Research has shown that the expression of certain pro-inflammatory cytokines is elevated in PCOS patients. Individuals with PCOS have higher levels of circulating inflammatory factors, including serum CRP,^[19]^ interleukin-6 ^[20]^, IL-17A,^[21]^ tumor necrosis factor-α,^[22]^ α-1 acid glycoprotein,^[23]^ monocyte chemoattractant protein-1,^[24]^ adipokines and their analogs,^[25]^ C1q, and TNF-related 6.^[26]^ Cytokines are secreted by leukocytes, oocytes, and follicular cells.^[18]^ These molecules regulate various aspects of ovarian function through autocrine and paracrine mechanisms, including gonadal steroid synthesis, folliculogenesis, steroidogenesis, luteogenesis, ovarian cell proliferation, oogenesis, hormonal balance, and luteal function.^[18]^ Moreover, there is evidence suggesting the presence of cytokines in follicular fluid, which correlates with assisted reproductive techniques, oocyte quality, and embryonic development.^[27]^

However, the causal relationship between cytokines and PCOS and the underlying mechanisms remain unclear. Our study employed a comprehensive set of cytokine-related SNPs with identified sites and the largest PCOS GWAS database for the discovery of MR, complemented by substantial PCOS GWAS data for replication MR, to evaluate the causal effect of cytokines on PCOS. Collectively, our MR analysis revealed an inverse correlation between CXCL11, CD6, and CCL4 levels and the risk of PCOS.

Currently, there is limited research on the role of CXCL11 and CD6 in PCOS pathogenesis. The only available observational study by Hatziagelaki et al reported that serum levels of CXCL11 and CD6 in PCOS patients were closely associated with sex hormones but lacked comparison with non-PCOS women.^[28]^

CXCL11, also known as interferon-γ-inducible T-cell alpha chemoattractant, is a cytokine belonging to the CXC chemokine family. It consists of 94 amino acids.^[29]^ CXCL11 primarily functions as an inflammatory chemotactic factor by binding to its receptors, CXCR3 and CXCR7, on immune cells, such as activated T cells, which attract them to the site of injury.^[30]^ Initially thought to be a specific agonist of CXCR3, it was later found to CXCL11 exhibits antimicrobial activity and antagonize CCR3. Petkovic et al^[31]^ demonstrated that CXCL11 is a natural antagonist of CCR5. When interacting with CCR5, CXCL11 inhibits the activity of this receptor, thereby counteracting the effects of the pro-inflammatory chemokines MIP-1α and monocyte chemoattractant protein-1β (MIP-1β), which primarily exert their effects through CCR5 instead of CCR3.^[31]^

In previous observational studies, upregulation of CCR5 expression was observed in the peripheral blood mononuclear cells of patients.^[32]^ Additionally, letrozole-induced PCOS mouse models showed upregulation of CCR5 expression.^[33]^ Furthermore, serum levels of MIP-1α and MIP-1β were higher in PCOS patients than those in the control group.^[7]^

Our MR analysis revealed a causal relationship between increased CD6 expression and decreased risk of PCOS, whereas reverse MR analysis did not find any causal association between PCOS and CD6. CD6 is a type I transmembrane glycoprotein belonging to the ancient and highly conserved scavenger receptor cysteine-rich superfamily. Its primary expression is in T cells, where it interacts with CD166 (also known as activated leukocyte cell adhesion molecule, ALCAM)^[34]^ and CD318.^[35]^ CD6 functions as a versatile lymphocyte receptor that can modulate lymphocyte (T, B1a, NK) function either positively or negatively, depending on the intracellular signaling context. Although it has been implicated in multiple inflammatory diseases, its role of CD6 in PCOS pathogenesis, which is frequently linked to chronic low-grade inflammation, remains unclear. Considering that CD6 signaling serves as a hub for assembling multiple enzymes and adaptors that can have positive or negative effects on signal propagation in different stimulus environments, potentially leading to diverse immune response outcomes,^[36]^ further research, particularly focusing on potential mechanisms, is valuable for elucidating the potential role of CD6 in the development of PCOS.

The meta-analysis integrated evidence suggesting a causal link between increased CCL4 levels and increased PCOS risk in PCOS.

CCL4, also known as MIP-1β, is a T-helper1 (Th1)-type CC chemokine that is mainly produced by activated monocytes and is regulated by multiple cytokines. Evidence from previous observational studies was consistent with our MR analysis. For example, a case-control study involving 60 PCOS patients and 30 controls reported that serum levels of CCL4 were higher in PCOS patients compared to controls (191.85 ± 17.54 vs 165.31 ± 11.01; *P* > .05).^[7]^ Other studies have reported similar results, showing that MIP-1β concentrations in follicular fluid are increased in non-obese PCOS patients.^[37]^ Our genetic MR analysis investigated the causal relationship between CCL4 and PCOS, consistent with these findings. Our results suggest that elevated CCL4 levels may increase the risk of developing PCOS. However, the reverse MR analysis did not establish a direct causal relationship between PCOS and CCL4. A prospective cohort study has revealed that CCL4 expression influences the developmental competence of embryos, indicating its potential role in embryonic development. Previous MR studies investigating the causal relationship between cytokines and PCOS identified associations between IL-17, SDF1a, and IL-4.^[38]^ However, these associations were not confirmed in our primary and replication MR analyses, which included a large sample size of PCOS cases and the most extensive range of cytokines in GWAS to date. It also includes discovery MR analysis, replication MR analysis, and meta-analysis, to ensure robust results.

Notably, our analysis revealed a causal relationship between CXCL11, CD6, and PCOS, which has rarely been documented in literature. Further investigation is warranted to validate CXCL11 and CD6 as potential biomarkers for PCOS prevention and treatment.

Despite these advances, the present study had several limitations. First, similar to other studies, the number of exposure SNPs of interest at the whole-genome level was limited. To address this, slightly relaxed thresholds were applied to MR analysis, which may have introduced false positives. However, in our MR analysis, the *F* statistics of the selected SNPs exceeded 10, indicating the robustness of the instrumental variables. Second, the study was based on samples of European ancestry, necessitating validation of the results across different ethnicities. Third, the types of cytokines examined are limited. Future studies should expand the scope of their analyses to enhance the comprehensiveness of their conclusions. Additionally, PCOS data were derived from female participants, whereas cytokine data included both male and female participants. Although this study proposed new potential associations through MR analysis, these findings require further confirmation through randomized controlled trials and basic research.

## 5. Conclusion

Our MR study indicated a potential causal relationship between the 3 circulating cytokines (CXCL11, CD6, and CCL4) and the altered risk of PCOS. Further research is required to validate these findings, investigate their underlying biological mechanisms, and assess their potential as therapeutic targets.

## Acknowledgments

The authors would like to thank Paperpal for providing language editing and proofreading support, which significantly enhanced the clarity and quality of this manuscript.

## Author contributions

**Data curation:** Yumin Jiang, Yuhua Huang, Yunqing Li.

**Conceptualization:** Yunqing Li.

**Formal analysis:** Yuhua Huang, Yunqing Li.

**Funding acquisition:** Yuhua Huang.

**Investigation:** Yumin Jiang.

**Methodology:** Yumin Jiang, Yuhua Huang, Yunqing Li.

**Software:** Yunqing Li.

**Supervision:** Yuhua Huang.

**Validation:** Yumin Jiang, Yuhua Huang.

**Visualization:** Yumin Jiang, Yunqing Li.

**Writing – original draft:** Yumin Jiang, Yunqing Li.

**Writing – review & editing:** Yumin Jiang, Yuhua Huang, Yunqing Li.

## Supplementary Material


