# Supplementary material for: Circulating cytokines levels and the risk of polycystic ovary syndrome: A Mendelian randomization analysis
Source: Medicine (Baltimore). 2025 Feb 28;104(9):e41359. doi: 10.1097/MD.0000000000041359 (PMC11875618; doi:10.1097/MD.0000000000041359)

Supplementary Figure 1. Discovery MR leave-one-out sensitivity analysis for cytokinesis on polycystic ovary syndrome(PCOS)

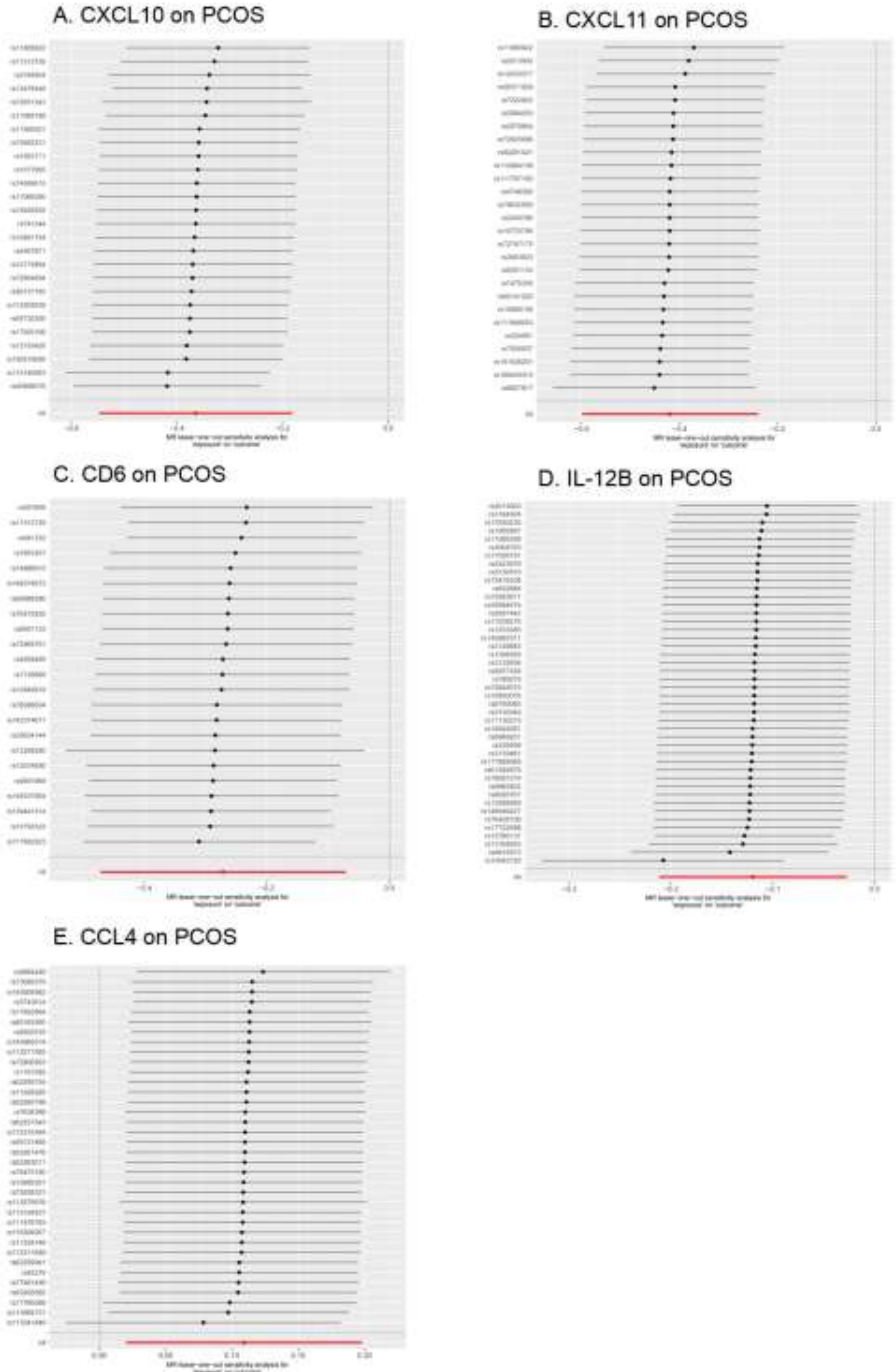

Supplementary Figure 2. Replicate MR leave-one-out sensitivity analysis for cytokinesis on polycystic ovary syndrome(PCOS)

A. CXCL6 on PCOS

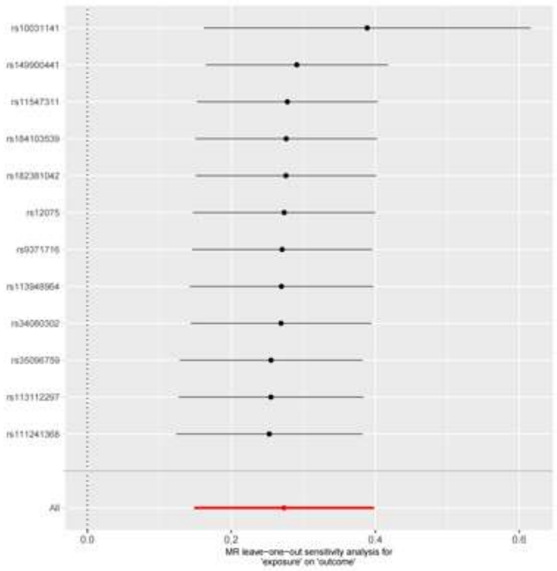

B. CCL4 on PCOS

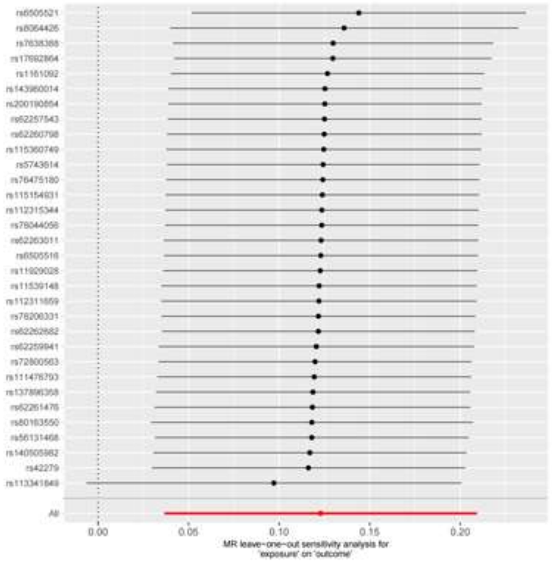

Supplement: Supplementary file 2 [file medi-104-e41359-s002.pdf]
